# Home modification to reduce falls at a health district level: Modeling health gain, health inequalities and health costs

**DOI:** 10.1371/journal.pone.0184538

**Published:** 2017-09-14

**Authors:** Nick Wilson, Giorgi Kvizhinadze, Frank Pega, Nisha Nair, Tony Blakely

**Affiliations:** BODE^3^ Programme, Department of Public Health, University of Otago, Wellington, New Zealand; Scientific Institute of Public Health (WIV-ISP), BELGIUM

## Abstract

**Background:**

There is some evidence that home safety assessment and modification (HSAM) is effective in reducing falls in older people. But there are various knowledge gaps, including around cost-effectiveness and also the impacts at a health district-level.

**Methods and findings:**

A previously established Markov macro-simulation model built for the whole New Zealand (NZ) population (Pega et al 2016, *Injury Prevention*) was enhanced and adapted to a health district level. This district was Counties Manukau District Health Board, which hosts 42,000 people aged 65+ years. A health system perspective was taken and a discount rate of 3% was used for both health gain and costs. Intervention effectiveness estimates came from a systematic review, and NZ-specific intervention costs were extracted from a randomized controlled trial.

In the 65+ age-group in this health district, the HSAM program was estimated to achieve health gains of 2800 quality-adjusted life-years (QALYs; 95% uncertainty interval [UI]: 547 to 5280). The net health system cost was estimated at NZ$8.44 million (95% UI: $663 to $14.3 million). The incremental cost-effectiveness ratio (ICER) was estimated at NZ$5480 suggesting HSAM is cost-effective (95%UI: cost saving to NZ$15,300 [equivalent to US$10,300]). Targeting HSAM only to people age 65+ or 75+ with previous injurious falls was estimated to be particularly cost-effective (ICERs: $700 and $832, respectively) with the latter intervention being cost-saving. There was no evidence for differential cost-effectiveness by sex or by ethnicity: Māori (Indigenous population) vs non-Māori.

**Conclusions:**

This modeling study suggests that a HSAM program could produce considerable health gain and be cost-effective for older people at a health district level. Nevertheless, comparisons may be desirable with other falls prevention interventions such as group exercise programs, which also provide social contact and may prevent various chronic diseases.

## Introduction

Various interventions that aim to prevent injurious falls in older people, include: exercise interventions, medication review and home safety assessment and modification (HSAM) [1] [2] [3]. This latter intervention involves a personalized assessment of injury hazards in the home (generally by an occupational therapist), followed by the systematic removal of these hazards [4]. The latter includes removing tripping hazards, adding grab bars in bathroom and toilet areas, adding hand rails to stairways, and improving home lighting [4].

A systematic review by Chase et al in 2012 [5] reported “moderate” evidence for falls prevention from HSAM (based on four studies). Similarly, a Cochrane systematic review [1] identified a reduction in significant reduction in falls (risk ratio (RR) = 0.81, 95% CI: 0.68 to 0.97; six trials; 4208 participants), but not specifically in *injurious* falls. A subsequent meta-analysis of six studies in 2013 [6], also suggested that HSAM significantly reduces falls. But since these reviews were published there is more evidence in the form of a randomized community trial [7]. It found that the HSAM intervention did significantly reduce *injurious* falls by 26% or by 39% when considering injuries specific to the home-modification intervention (RR = 0.61, 0.41–0.91). However, this study was for all age-groups and not just older people.

A systematic review has also identified seven health economic studies of the HSAM intervention [8]. It found mixed evidence for cost-effectiveness, albeit with a majority of studies tending to suggest that HSAM was cost-effective or even cost-saving. Subsequently published work also suggests relative cost-effectiveness [9] or HSAM being cost-saving (e.g., when health gain benefits are monetized [10]).

Given this background we aimed in the study presented here to further explore the likely cost-effectiveness of the HSAM intervention–but to do so at a local health district board (DHB) level (a level of the health system which had not been studied before to our knowledge). In particular, we aimed to determine how a DHB might optimally target the HSAM intervention (e.g., by demographic factors and risk factors such as previous falls) so as to potentially: maximize health gain, maximize health inequalities reductions, minimize health costs (for treating injurious falls), and minimize upfront intervention costs for the DHB. We also considered the major drivers of uncertainty around the HSAM intervention–which could then help prioritize any further research efforts relating to the use of this intervention at a DHB level.

Accordingly, the specific aims of this study were: (i) to estimate the health gain, health inequality impacts and cost-utility from a health district-level program of HSAM for reducing injurious falls in older people using an enhanced version of an established model; (ii) how targeting the intervention by age and previous fall history may alter cost-effectiveness; and (iii) to identify the most uncertain model parameters that might justify further research to enhance certainty for decision-making.

### Background on the health district and New Zealand context

The district of Counties Manukau DHB (CMDHB) was selected as its leadership has expressed to us an interest in such modeling work and it is characterized by having relatively high levels of deprivation, relatively high populations of Māori (Indigenous) and Pacific peoples, and relatively high levels of household crowding. It comprises 11.1% of the total New Zealand population, with 10.7% of its population in the 65+ year age-group (n = 50,247 people). While most of the CMDHB population lives in urban areas, some is also rural (see a map of the boundaries of CMDHB here: http://www.health.govt.nz/system/files/documents/pages/counties-manukau.pdf). In terms of injurious falls in the 65+ year population of CMDHB, there were 1364 hospitalized fall-related injuries and 5097 non-hospitalized fall-related injuries in 2011 (Accident Compensation Corporation [ACC] [11] data provided to the authors). The proportion of these falls in Māori was relatively low (4.3% of all non-hospitalized falls and 5.1% of all hospitalized falls), a pattern likely to be due to the younger age structure of the Māori population. Nevertheless, we have considered analyses by ethnicity in this study since this is routine for the New Zealand health sector as part of considering all ways in which substantive ethnicity health inequalities in this country might be addressed.

The cost of all these falls in the 65+ age-group in CMDHB was NZ$7.30 million in 2011 (ACC data). While the CMDHB is compensated for these costs by ACC, there are additional costs borne just by the CMDHB given that bed capacity can often limited (especially in winter months) if there are delays in moving patients from hospital to residential care facilities. This means that the ability of the DHB to reduce waiting lists for surgical interventions may be constrained.

The wider New Zealand context is that falls are an important cause of morbidity and mortality among New Zealanders aged 75+ years (the seventh most important cause at 3.1% of all disability-adjusted life-years [DALYs]) [12]. Furthermore, data for the 2014 financial year in New Zealand shows that falls in older adults cost NZ$159 million in claims to the national injury insurer, the ACC. In response to such impacts, the New Zealand Government has scaled up its investment in falls prevention in mid-2016 [13].

## Methods

### Perspectives and general approach

We adapted and re-parameterized the existing BODE^3^ Falls Prevention Model that had been designed to study falls prevention at a national level [9]. In summary, the design of this model followed the BODE^3^ Protocol [14] and took a health system perspective i.e., evaluating just health gains and health system costs for the rest of the lives of the modeled cohort (and not wider societal benefits and costs such as impacts on productivity in older people still working and in terms of pension payments to older people). It used QALYs as the outcome measure with these derived from a New Zealand burden of Disease Study (see Protocol [14]), and disability weights from the Global Burden of Disease Study [15].

The target population for the HSAM intervention was community-dwelling older people aged 65+ years in the year 2011 who were resident in the CMDHB Health District. The model followed up the entire simulated population either to death or to 110 years of age. We assessed the effect of HSAM on injurious falls in the home leading to any health service use, but not on outcomes with less-quantifiable impacts on QALYs (e.g., fear from injurious falls). In the base-case analysis, we compared targeted HSAM with no intervention, which can be regarded as current “business-as-usual” in New Zealand and CMDHB. The latter is a reasonable assumption given that no such HSAM programs have been established and local data indicate nearly all New Zealand homes can potentially benefit from fall-preventing modifications [7]. In scenario analyses, the analysis was expanded in a range of ways e.g., to targeting those with a previous injurious fall and by age-group (e.g., 75+ years), and by time period into the future. In the base-case analysis, the standard discount rate of 3% was applied to both QALYs gained and costs. In scenario analyses, we used 0% and 6%, respectively, for both health gains and costs.

### Core model structure

As per most previous studies on this topic we used a Markov macro-simulation model with annual cycles (see Figure A in S1 Appendix and Pega et al [9]). The model commenced with the target cohort of community-dwelling older people aged 65+ years starting in a non-injured state in 2011. The model estimated the effect of HSAM on QALYs gained, net health system costs and cost-effectiveness in the target population by modeling the reduction of injurious falls in the home (and the associated burden of disease and costs, respectively).

We modeled heterogeneity in the incidence rates of injurious falls by age-group (65–69, 70–74, 75–79, 80–84, 85+), sex, and ethnicity (Māori and non-Māori). The model also considered two risk groups, “low risk” for people with no previous treatment for injurious falls and “high risk” for people with a previous injurious fall requiring treatment (in the preceding five years), and modeled the effect of HSAM on injurious falls (Figure A in S1 Appendix). The modeled people could either have or not have an injurious fall event, with injured fallers either requiring non-hospital health care or hospitalization. At each point, a person could move into a residential aged care facility, whereby the benefit of the community-based HSAM intervention for them ceased.

### Model parameters

The key demographic, epidemiological, intervention and cost model parameters are presented in Table 1. Some were specific to the CMDHB, but others were based on national level data as appropriate.

**Table 1:** Demographic, epidemiological, intervention and cost parameters for modeling the home safety assessment and modification (HSAM) intervention for preventing falls in a health district population (Counties Manukau District Health Board [CMDHB]).

Parameter	Source/s	Selected values (as appropriate)	Trends, uncertainty and additional details. (Standard BODE^3^ methods were used. Extra detail is generally included in the BODE^3^ Study Protocol [14])
***Demographic and dwelling characteristics***			
Population (aged 65+) in CMDHB.	Statistics New Zealand (SNZ) estimates for 2011 by sex, age-group and ethnicity.	41,736	The population in CMDHB for 2011 was interpolated from the 2006 and 2013 censuses. *Trend*: No cohort replenishment over time (closed cohort).
Annual probability of moving into residential aged care facilities (%) (national level data).	New Zealand (NZ) Census of Population and Dwellings 2013.	Range: 0% to 2% (varies by age-group).	The people who transition to residential aged care facilities were treated as no longer potentially being able to benefit from the HSAM intervention. *Uncertainty*: Log-normal with scalar (0.5 to 1.5 of the point estimate). For extra details see the Appendix in Pega et al 2016 [9].
Annual probability of moving house (%) in the 65+ age-group (national level data).	NZ Census of Population and Dwellings 2013.	Range: 2% to 5% (varies by population group).	This variable captures movement out of modified houses, and then subsequent movement into unmodified or modified houses. *Uncertainty*: Log-normal with scalar (0.5 to 1.5 of the point estimate). For extra details see the Appendix in Pega et al 2016.
Level of dwelling cohabitation (impacts on intervention costs).	NZ Census of Population and Dwellings 2013.	Occupancy was higher in CMDHB vs nationally (3.21 vs 2.67 per dwelling).	If two people aged 65+ resided in the same dwelling then this halved the cost of applying the HSAM intervention (on a per participant basis). For targeted interventions, the oldest resident of the pair was the one considered around HSAM intervention eligibility.
***Injury epidemiology characteristics***			
Annual probability of injurious fall (%) for CMDHB by age-group, sex and ethnicity.	Accident Compensation Corporation (ACC) claims registry data provided to BODE^3^ (including falls that only involve treatment in primary care)	15.5% overall (6461/41,736) for 2011.	The claims data should be relatively robust for all injuries requiring treatment in NZ as the health system is highly motivated to make claims to ACC since this ensures payment by ACC. The CMDHB data were adjusted by national level data to provide low and high risk group distributions (the latter if having had treatment for an injurious fall in the previous five years). In scenario analysis the HSAM intervention was targeted by risk level (and also by age-group). *Uncertainty*: Log-normal with scalar (0.5 to 1.5 of the point estimate). For examples of national level probabilities by age-group, see the Appendix in Pega et al 2016.
Probability of hospitalization after an injurious fall (%) for CMDHB	ACC claims registry data provided to BODE^3^.	3.3% overall (1364/41,736) for 2011.	As above, the claims data should be relatively robust for all injuries requiring hospitalization in NZ as ACC pays DHBs for the costs of treatment. *Uncertainty*: Log-normal with scalar (0.5 to 1.5 of the point estimate). For examples of national level probabilities by age-group, see the Appendix in Pega et al 2016.
Probability of death after an injurious fall (%) (national data).	ACC claims registry data provided to BODE^3^.	Range: 0% to 5% (highest in Māori men aged 65–69).	*Uncertainty*: Log-normal with scalar (0.5 to 1.5 of the point estimate). For extra details see the Appendix in Pega et al 2016.
Morbidity from falls (disability weights) used to determine QALY loss.	Based on Global Burden of Disease (GBD) data [15].	Disability weight = 0.10 (annualized).	*Uncertainty interval (95%)*: 0.06 to 0.15. We assumed that each injurious fall accrued the disability weight [DW] for a fracture of 0.30 which was applied for a four-month period over the one year cycle. We acknowledge this is a simplifying assumption that does not capture the heterogeneity of different injuries that occur, but we were focused on injuries captured in official data and which were likely to be associated with medical attention (e.g., DWs from the GBD for a sprain [0.009] might not always receive medical attention). But the following would almost certainly get such attention: clavicle/scapula/humerus fracture [DW = 0.053], neck of femur fracture [0.308], and for a fractured pelvis [0.39]. Also, we did not include accounting for long-term (post one-year) disability–even though this may be large in some cases (e.g., 0.194 for fractured pelvis). We also did not consider anxiety associated with fear of falling (which appears to be modified by some fall prevention interventions [16]).
***Background epidemiological characteristics***			
All-cause mortality rates	SNZ mortality rates by age, sex and ethnicity (national level data)	–	*Trends*: Annual declines of 2.25% and 1.75% were modeled for Māori and non-Māori all-cause mortality rates respectively (see BODE^3^ Protocol [14] and related work [17]). Trends were modeled out to 2026 with 0% per annum decline for both ethnic groupings thereafter. *Uncertainty*: Nil.
Total morbidity per capita in 2011	The per capita rate of years of life lived with disability (YLD) from the NZ Burden of Disease Study by age, sex and ethnicity (national level) [14].	–	*Trend*: Nil (i.e., assumed constant into the future). *Uncertainty*: Nil.
***Intervention***			
Assumed effectiveness of HSAM	Cochrane systematic review of interventions for preventing falls in older people [1].	19% reduction in the rate of injurious falls	Since evidence on the effectiveness of HSAM on *injurious* falls is still uncertain in the older age-group (albeit there is favorable evidence from a randomized control trial (RCT) for all age-groups [7]), we assumed that HSAM reduces the rate of falling as per the Cochrane review (risk ratio (RR) = 0.81, 95% CI: 0.68 to 0.97; six trials; 4208 participants) [1], to the same degree as it reduces the rate of *injurious* falling. (Indeed a majority of reported falls do cause injury e.g., 68% in one study [18].) *Uncertainty*: 95% confidence interval (CI): 3%-32% [1]. We assumed the parameter to have a log-normal distribution. *Heterogeneity*: The same level of effectiveness was applied to all population groups. *Trend*: No trend in declining effectiveness of the intervention was assumed given that most home modifications are fairly robust (e.g., grab rails). But we included a scenario analysis around declining intervention effectiveness over 10 years (linearly decreasing to nil).
Intervention uptake in CMDHB	A NZ-based RCT of HSAM [7].	89.0% uptake (as per the RCT at 842/946 households participating)	*Uncertainty*: 95%CI: 64% to 99% (beta distribution with alpha = 10.03, beta = 1.24). The uncertainty was set at a relatively high level as the trial population covered household residents of all ages and this population were relatively deprived (Community Services Card holders). That is, these eligibility cards indicate that the person is entitled to state subsidies and are held by people on a relatively low income, unemployed individuals, students, pensioners (age 65 years or older), and people in receipt of sickness benefits.
***Costs (all NZ$ in the 2011 year)***			
Program running costs for CMDHB	Informed by a NZ pilot cancer screening program cost data [19].	$1,836,384	The program was assumed to run like a screening program where participants are “screened” for their agreement to participate (have their home modified). That is each person aged 65+ is sent a letter by DHB staff and there is a follow-up phone call (to determine participation and if so to arrange a time for the HSAM). The invitation cost from the pilot screening program data was $44 per participant which was multiplied by the DHB eligible population aged 65+ to give the total program cost ($44 x 41,736 = $1,836,384). The targeted interventions in scenario analyses used the same $44 value per person but had different target populations (e.g., for those aged 75+ with a history of falls). *Uncertainty*: SD of +/-10% of the point estimate (of $44), gamma distribution.
HSAM intervention costs	A NZ-based RCT of HSAM [7].	Intervention cost per person $250	From the RCT we extracted cost data (i.e., labor and material costs) for indoor components of the HSAM in households with one or more members aged 65 years or over. *Uncertainty (95%UI)*: $165 to $355, in 2011 values [7]. *Adjustments*: The modeling used a per participant cost so this was influenced by dwelling cohabitation (see above). In scenario analyses for targeting the high risk groups who developed a history of treatment for an injurious fall–then intervention costs occurred at various times in the future. In a scenario analysis we considered the possibility of a DHB negotiating with a single provider of HSAM services and so achieving likely economies of scale (i.e., a hypothetical one third reduction in costs).
Costs of non-hospital health care after falling (i.e., in primary health care) for CMDHB	ACC claims registry data provided to BODE^3^.	Average: $344 per case (but varies by age and sex)	*Uncertainty*: Log-normal with scalar (0.5 to 1.5 of the point estimate). There is full coverage by ACC of fall-related health costs so we did not include an out-of-pocket cost component for citizens. Examples of these costs at the national level are given in the Appendix of Pega et al 2016.
Costs of hospitalization after falling for CMDHB	ACC claims registry data provided to BODE^3^.	Average: $4068 per case (but varies by age and sex)	*Uncertainty*: Log-normal with scalar (0.5 to 1.5 of the point estimate). Hospital care if provided for free in NZ, albeit with hospitals billing ACC for injury costs. Examples of these costs at the national level are given in the Appendix of Pega et al 2016.
Annual average population health system costs by age-group and sex (national costs)	HealthTracker [20]	Range for not in the last 6 months of life: $3378 in women aged 65-69y, to $6511 in men aged 85-89y. Range in the last 6 months of life: $6127 in women aged 95-99y to $20,476 in women aged 65-69y.	*Uncertainty*: Log-normal with scalar (0.5 to 1.5 of the point estimate). *Heterogeneity*: Only by age and sex. HealthTracker data had to be scaled (by 1.21) to account for residual limitations with the comprehensiveness of national data. Proximity to death was accounted for (i.e., costs in the last six months of life) with these being scaled by 1.1 to 1.3 depending on age-group (to account for national data not containing certain end-of-life costs). A BODE^3^ Programme upgrade in costing methods in mid-2016 has resulted in lower costs in the last six months of life compared to previous modeling work.

### Analysis

The model was built in TreeAge Pro version 2013. Monte Carlo simulation with 2000 iterations was used, generating an output uncertainty interval about the QALYs gained, net cost and ICERs, arising from probabilistic sampling from the uncertainty about each input parameter (Table 1) in each iteration. To assess what ‘drives’ uncertainty in each of the QALYs, net cost and ICER outputs, we reran the model for the 2.5^th^ and 97.5^th^ percentile of each input parameter’s uncertainty distribution (one by one), and plotted the results as tornado plots.

## Results

### Base-case analyses

In the 65+ age-group in this local health district, the HSAM program was estimated to achieve health gains of 2800 QALYs over the remaining life time of this cohort (95% uncertainty interval [UI]: 547 to 5280) (Table 2). The estimated health gain per capita in the 65+ year population was 0.066 QALYs (Table 3) which is equivalent to an extra 24 days of healthy life, although this would be slightly higher in those who participated by allowing the HSAM intervention in their homes.

**Table 2:** Main results and scenario analyses for the home safety assessment and modification intervention (HSAM) in a single health district (Counties Manukau District Health Board).

Intervention/Output	Output	Baseline (no HSAM)*	Impact of HSAM compared to baseline*
***Base-case intervention (3% discount rate)***			
Net cost (NZ$; 1000s)	Mean	$3,040,000	$8440
	95%UI	($3,010,000 –$3,110,000)	($663 –$14,300)
QALYs gained	Mean	331,000	2800
	95%UI	(321,000–342,000)	(547–5280)
ICER	Mean	Not applicable (NA)	$5480
	Median	NA	$3310
	95%UI	NA	(cost saving–$15,300)
***HSAM targeted to “at risk” people aged 65+ years with one or more previous injurious falls***			
Net cost ($; 1000s)	Mean	$3,040,000	$91
	95%UI	($3,010,000 –$3,110,000)	(cost saving–$2140)
QALYs gained	Mean	331,000	1420
	95%UI	(321,000–342,000)	(258–2760)
ICER	Mean	NA	$701
	Median	NA	$331
	95%UI	NA	(cost saving–$2740)
***HSAM targeted to people aged 65+ years but with no history of previous injurious falls***			
Net cost ($; 1000s)	Mean	$3,040,000	$8340
	95%UI	($3,010,000 –$3,110,000)	($3770 –$12,800)
QALYs gained	Mean	331,000	1520
	95%UI	(321,000–342,000)	(292–2940)
ICER	Mean	NA	$9600
	Median	NA	$5790
	95%UI	NA	($1410 –$26,300)
***HSAM targeted to people aged 75+ years***			
Net cost ($; 1000s)	Mean	$963,000	$2430
** **	95%UI	($951,000 –$987,000)	(cost saving–$4830)
QALYs gained	Mean	85,700	592
** **	95%UI	(83,800–87,800)	(116–1120)
ICER	Mean	NA	$8490
** **	Median	NA	$4620
** **	95%UI	NA	(cost saving–$24,500)
***HSAM targeted to “at risk” people aged 75+ years with one or more previous injurious falls***			
Net cost ($; 1000s)	Mean	$963,000	$-163
	95%UI	($951,000 –$987,000)	(cost saving–$484)
QALYs gained	Mean	85,700	281
	95%UI	(83,800–87,800)	(52–546)
ICER	Mean	NA	$832
	Median	NA	$-87
	95%UI	NA	(cost saving–$4604)
***HSAM targeted to people aged 75+ years but with no history of previous injurious falls***			
Net cost ($; 1000s)	Mean	$963,000	$2540
	95%UI	($951,000 –$987,000)	($282 –$4420)
QALYs gained	Mean	85,700	338
	95%UI	(83,800–87,800)	(64–648)
ICER	Mean	NA	$14,260
	Median	NA	$8020
	95%UI	NA	($206 –$40,200)
***Discount rate 0% (otherwise same as base-case model)***			
Net cost ($; 1000s)	Mean	$4,070,000	$14,000
** **	95%UI	($4,050,000 –$4,130,000)	($7160 –$21,200)
QALYs gained	Mean	427,000	4160
** **	95%UI	(412,000–444,000)	(813–7860)
ICER	Mean	NA	$5030
** **	Median	NA	$3560
** **	95%UI	NA	($1170 –$11,600)
***Discount rate doubled to 6% (otherwise same as base-case model)***			
Net cost ($; 1000s)	Mean	$2,390,000	$6110
	95%UI	($2,360,000 –$2,460,000)	(cost saving–$12,300)
QALYs gained	Mean	270,000	2010
	95%UI	(262,000–277,000)	(392–3830)
ICER	Mean	NA	$6440
	Median	NA	$3420
	95%UI	NA	(cost saving–$20,100)
***HSAM targeted to at risk older people aged 65+ years with one or more previous injurious falls with declining HSAM intervention effectiveness over 10 years***			
Net cost ($; 1000s)	Mean	$3,040,000	$9430
** **	95%UI	($3,014,000 –$3,110,000)	($5600 –$13,000)
QALYs gained	Mean	331,000	1230
	95%UI	(321,000–342,000)	(222–2470)
ICER	Mean	NA	$12,900
** **	Median	NA	$8100
** **	95%UI	NA	($2600 –$38,900)
***HSAM targeted to at risk older people aged 65+ years with home modification costs hypothetically reduced by one third (funder achieves economies of scale with purchasing interventions)***			
Net cost ($; 1000s)	Mean	$3,040,000	$5860
** **	95%UI	($3,010,000 –$3,110,000)	(cost saving–$10,900)
QALYs gained	Mean	331,000	2800
** **	95%UI	(321,000–342,000)	(547–5280)
ICER	Mean	NA	$3920
** **	Median	NA	$2390
** **	95%UI	NA	(cost saving–$11,600)
***Base-case model but with just a 10 year time horizon for benefits and costs***			
Net cost ($; 1000s)	Mean	$2,050,000	$3290
	95%UI	($2,014,000 –$2,120,000)	(cost saving–$10,600)
QALYs gained	Mean	247,000	1380
	95%UI	(241,000–252,000)	(264–2760)
ICER	Mean	NA	$7220
	Median	NA	$2940
	95%UI	NA	(cost saving–$29,700)
***Base-case model but with just a 20 year time horizon for benefits and costs***			
Net cost ($; 1000s)	Mean	$2,830,000	$4950
	95%UI	($2,790,000 –$2,910,000)	(cost saving–$11,800)
QALYs gained	Mean	315,0000	2360
	95%UI	(306,000–324,000)	(466–4540)
ICER	Mean	NA	$4980
	Median	NA	$2470
	95%UI	NA	(cost saving–$17,200)

Note

*Results are rounded to three meaningful digits.

ICER: incremental cost-effectiveness ratio; QALY: quality-adjusted life-year; 95%UI: 95% uncertainty interval.

**Table 3:** Analyses by ethnicity and sex within the base-case model for the home safety assessment and modification (HSAM) intervention in the Counties Manukau District Health Board (95%UI).

Population group	Baseline (no HSAM)*			HSAM compared to baseline (incremental)*			
	Net cost ($; 1000s)	QALYs expected	QALYs expected per capita	Net cost ($; 1000s)	QALYs gained	QALYs gained per capita	ICER
Total population	$3,040,000 ($3,010,000 –$3,110,000)	331,000 (321,000–342,000)	7.94 (7.69–8.20)	$8440 ($663 –$14,300)	2800 (547–5280)	0.066 (0.013–0.126)	$5480 (cost saving–$15,300)
Māori (Indigenous population)	$173,000 ($172,500 –$175,000)	17,100 (16,500–17,700)	6.61 (6.36–6.84)	$900 ($512 –$1310)	158 (29–307)	0.061 (0.011–0.118)	$8360 ($2370 –$21,000)
Māori: equity analysis**	$196,000 ($195,000 –$198,000)	22,700 (21,800–23,700)	8.75 (8.39–9.11)	$1140 ($618 –$1700)	240 (45–458)	0.092 (0.017–0.176)	$6540 ($2490 –$14,700)
Non-Māori	$2,870,000 ($2,840,000 –$2,930,000)	314,000 (304,000–325,000)	8.031 (7.78–8.29)	$7540 (cost saving–$13,100)	2640 (514–4970)	0.067 (0.013–0.126)	$5310 (cost saving–$15,100)
Men	$1,460,000 ($1,450,000 –$1,500,000)	147,000 (142,000–153,000)	7.65 (7.38–7.92)	$4020 (cost saving–$7030)	1330 (256–2560)	0.068 (0.013–0.132)	$5460 (cost saving–$16,900)
Men: equity analysis***	$1,600,000 ($1,580,000 –$1,640,000)	163,000 (157,000–169,000)	8.45 (8.13–8.78)	$4770 ($726 –$7970)	1580 (306–3010)	0.082 (0.015–0.155)	$5070 ($159 –$14,500)
Women	$1,580,000 ($1,570,000 –$1,610,000)	184,000 (179,000–190,000)	8.20 (7.96–8.44)	$4420 ($654 –$7330)	1470 (291–2760)	0.065 (0.012–0.122)	$5520 ($165 –$14,900)

Notes

* Results are rounded to three meaningful digits.

**As Māori have higher background mortality rates and higher morbidity, this essentially ‘penalises’ health gain for Māori in the standard analyses. So we present an equity analysis [21] with non-Māori morbidity and mortality rates applied to Māori (i.e., expanding the envelope of potential health gain for those benefiting from the HSAM intervention).

*** As men have higher background mortality rates, this essentially ‘penalises’ health gain for men in the analyses. So we present an equity analysis with women’s morbidity and mortality rates applied to men.

ICER: incremental cost-effectiveness ratio; QALY: quality-adjusted life-year; 95%UI: 95% uncertainty interval.

The one-off intervention cost for running the program and assessing and modifying all the dwellings was estimated at $9.57 million [m] (Table 4). The net health system costs comprised: intervention costs, healthcare costs saved from falls prevented, and additional healthcare costs from extra years of life lived. These estimated net health system costs for the remaining lives of the modeled cohort were $8.44m (95%UI: $663 to $14.3m). The incremental cost-effectiveness ratio (ICER) was $5480 (95%UI: cost saving to $15,300), suggesting HSAM is very cost-effective. For this assessment our research program routinely uses a cost-effectiveness threshold based on the WHO approach of using the gross domestic product per capita of a country (which for NZ is $45,000 per QALY for 2011 [22], with additional explanations elsewhere [23]). This approach is used, given the absence of an agreed cost-effectiveness threshold for New Zealand Government agencies.

**Table 4:** Intervention costs by population group offered the home safety assessment and modification (HSAM) intervention (age-group and injurious fall history).

Population group offered the HSAM intervention	Intervention costs covering the program administration costs and HSAM costs in NZ$ (95% uncertainty interval)
People aged 65+ years	$9.57 million [m] ($6.40m –$13.3m)
“At risk” people aged 65+ years with one or more previous injurious falls	$864,000 ($384,000 –$1.48m)
People aged 65+ years but with no history of previous injurious falls	$8.70m ($5.84m –$12.1m)
People aged 75+ years	$3.67m ($2.46m –$5.09m)
“At risk” people aged 75+ years with one or more previous injurious falls	$416,000 ($185,000 –$711,000)
People aged 75+ years but with no history of previous injurious falls	$3.26m ($2.18m –$4.54m)

### Population group and equity analyses

For the base-case intervention of HSAM in the 65+ population, the health gain and cost-effectiveness were fairly similar for women and men, and for the Māori and non-Māori populations (Table 3). When performing an “equity analysis”, Māori gained an extra 26% of QALYs per capita compared to non-Māori and men gained an extra 21% of QALYs per capita compared to women–but the 95% uncertainty intervals overlapped substantially (see Table 3 footnotes for details).

### Scenario analyses

Setting the discount rate to 0% and 6% for the base-case intervention did not markedly impact on the ICERs ($5030 and $6440 respectively) given how health gain and costs were distributed over time (Table 2). Targeting HSAM only to people aged 65+ in the CMDHB District with previous injurious falls lowered upfront intervention costs (to $864,000, Table 4) and net health system costs (to $91,000) and further improved cost-effectiveness (ICER = $700 per QALY gained) (Table 2). But this resulted in lower total health gain (down to 1420 QALYs). In parallel, cost-effectiveness was less favorable for people aged 65+ in the CMDHB District *without* previous injurious falls (ICER = $9600 per QALY gained).

Targeting HSAM only to people aged 75+ years with previous injurious falls lowered intervention costs even further (to $416,000) and resulted in net cost savings to the health system ($163,000 in savings), but reduced total health gain (to 281 QALYs) and reduced cost-effectiveness ($832 per QALY gained) (Table 2). Other targeting scenarios were also cost-effective, but less so than the above i.e., all those aged 75+ (ICER = $8490), and all those 65+ with no history of falls (ICER = $9600).

If the structural changes from the HSAM intervention were assumed to decay over time with no residual benefit after 10 years, this reduced cost-effectiveness (ICER = $12,900). Yet if the service provider was able to reduce intervention costs by a third via economies of scale, then this would improve cost-effectiveness (ICER = $3920). A shorter time horizon of 10 years for considering health benefits and health costs, resulted in a reduced cost-effectiveness (ICER = $7220).

### Uncertainty analyses

Uncertainty analyses for the key model parameters for QALYs gained, incremental net costs and the ICER are shown in tornado plots for the base-case intervention (Figure B, Figure C, and Figure D in S1 Appendix). For QALYs gained, the major driver of uncertainty by far was the intervention effectiveness uncertainty for reducing the rate of injurious falls (Figure B in S1 Appendix; i.e., the range of low to high QALY gain for selecting the 2.5^th^ and 97.5^th^ percentile of input parameters was widest for the intervention effect–the top horizontal bar in the tornado plot). Likewise, this was also the major driver for the ICER (Figure D in S1 Appendix). For costs, the major driver was uncertainty about the probability of death from a fall, followed by the scaler for the cost of a hospitalized fall and the scaler for the probability of hospitalization once a fall had occurred (Figure C in S1 Appendix). Level of uptake was unimportant in determining the ICER (the 11^th^ ranked item).

## Discussion

### Main findings and interpretation

The HSAM intervention was estimated in this modeling work to produce considerable health gain and to be highly cost-effective among people aged 65+ years in this health district setting in a high-income country. Nevertheless, targeting HSAM to older people with previous injurious falls was estimated to reduce upfront intervention costs, as well as improving cost-effectiveness. Yet such targeting reduced total health gain obtained relative to the universal (all 65+) approach. Similarly, targeting the intervention to only those aged 75+ with previous injurious falls reduced upfront intervention costs and indeed was estimated to be cost-saving overall.

This modeling work has suggested that HSAM in this health district appears to be somewhat more cost-effective than previously estimated at the national level for New Zealand [9] (ICER = $5480 vs $9000). This difference is likely to reflect improvements in estimating health sector costs and differences associated with the district population (demographics, probability of injurious falls, and the higher level of dwelling co-habitation than nationally).

This modeling study found that all population groups (by ethnicity and sex) benefited from the HSAM intervention fairly much equally. This is probably not surprising given the background epidemiology of falls and because access to healthcare after falls is probably non-differential (since all such care is free from the patient perspective in New Zealand). This suggests that those policy-makers who are particularly focused on reducing health inequalities, might wish to prioritize other interventions (e.g., improving tobacco control and preventing obesity) ahead of introducing HSAM.

### Study strengths and limitations

As detailed for our previous work [9], the falls prevention model we used has a number of strengths. First, we modeled two distinct risk groups with their own fall rates, based on history of injurious falls, determined from the official national data sources (which contrasts to some previous models where expert opinion was relied on). Secondly, we were able to model heterogeneity by key population characteristics, and to provide an equity perspective for Māori vs non-Māori. Third, the study uses relatively high quality empirical data from national official registries to estimate the incidence of injurious falls, the associated health care utilization and the associated costs. Fourth, we were able to consider the issues of targeting HSAM to community-dwelling older people at high risk of injurious falling and providing the intervention prospectively over time. Also relative to our previous work we were able to make improvements to the previous model in terms of updated health cost data, and inclusion of additional parameters (e.g., level of uptake and program administrative costs).

Nevertheless, this modeling study has a number of limitations as itemized below.

- Given data limitations we were required to consider only *injurious* falls and to assume that HSAM is equally effective in reducing *injurious* falls as it is in reducing all falls (as reported by the Cochrane systematic review [1]). Yet it is plausible that HSAM has a greater or lesser effect on falls than on injurious falls in older people (though the trial performed for all age-groups [7] strongly suggests that HSAM does actually prevent injuries). Furthermore, it is possible that HSAM interventions actually reduce injury severity for injurious falls that still occur e.g., some partial use of a grab rail to slow a fall might mean that a minor fracture occurs rather than a major one (but data on this possibility were lacking). If this was the case then we have under-estimated the benefit of the HSAM intervention.
- As detailed in the Table 1, some of the model input parameters were based on assumptions that could be improved upon when additional data become available. For example, we can not be certain that the intervention uptake in the older population would be similar to the uptake in the low-income population involved in the New Zealand trial data for HSAM. While some older people may have high uptake (e.g., if they recognize they are at increased risk of falls due to their age), others may have lower uptake (if they consider it is not worth the trouble, intrusion or impact on house aesthetics).
- It is plausible that the effectiveness of the HSAM intervention varies by age-group though we did not have the data to model this specifically. For example, people in the older age-groups might be more likely to fall without the time to clasp hold of grab rails (e.g., falls from transient ischemic attacks or due to medication). But on the other hand, these older age-groups might be more likely to walk in closer proximity to grab rails, to routinely use railings on stairs, and have lower alcohol intakes.
- Aspects of the health gain and health system cost impacts were not captured given that benefits of the HSAM intervention did not consider falls reduction among younger people in the same household as an older person (or who subsequently moved into a modified house in the future). Indeed, in the New Zealand trial data 42% of the injurious falls in the control group were in people aged under 60 years [7]. The implication of this specific limitation alone is that the true cost-effectiveness of the HSAM intervention is probably more favorable (via greater health gain and greater health cost savings from reduced injurious falls). It might also mean that the intervention might be pro-equity given that low-income New Zealand families would be more likely to be in the same residence as older relatives who were being provided with the HSAM intervention.
- Future work could consider more fine-grained modeling of different types of fall-related injury (e.g., distinguishing wrist fractures vs hip fractures etc). Similarly, we did not consider the likely benefits of HSAM in terms of reduced anxiety around falling (given data limitations with measuring this impact).
- There remain various uncertainties around health costs in New Zealand [20], and further improvements are part of on-going work in our work program. In particular, background (non-fall-related) health costs could be improved upon since it is plausible that these costs are relatively higher in people who have injurious falls (i.e., certain co-morbid conditions that increase the risk of falls [24]).

This study used a health system perspective but wider perspectives would give different results. For example, a societal perspective would capture any economic benefits from keeping employed people aged 65+ years in the workforce or being able to contribute to the informal economy, such as care for grandchildren. It might also take into account the costs associated with the increased use of residential care after injurious falls, with one New Zealand study estimating that this was 41% of the costs from falls [25]. Finally, a wider perspective may also recognize that the HSAM intervention is not as costly as assumed in a health system perspective given that it may slightly improve the capital value of people’s homes (i.e., the government funded HSAM intervention modeled here involves a small wealth transfer to home owners from the government).

### Generalizability to other settings

It is likely that these results are generally applicable to other health districts in New Zealand and in similar countries internationally. Nevertheless, in some contexts the health gain might be greater if the resident population have a higher life expectancy than that for the studied DHB (i.e., there is a larger envelope of health gain from preventing fall-related mortality and morbidity). Intervention costs will also vary by the quality of the existing housing stock and the extent to which the 65+ population are in shared dwellings. Settings with large populations may also benefit from the economies of scale for purchasing HSAM interventions from service providers.

Intervention costs are possibly lower in New Zealand, at least compared to other OECD countries. On the other hand, as health costs in New Zealand are also relatively low, this might mean that in other high-income countries the savings in healthcare costs would be larger.

### Possible research and policy implications

Given there is still some residual uncertainty around the effectiveness of HSAM in reducing *injurious* falls in older people (the best trial evidence is for all age-groups collectively [7]), policy-makers may still wish to invest in more research around the effect size, since it remains the major driver of uncertainty in the results (Figure B in S1 Appendix). Other research could consider obtaining data on the extent to which the HSAM intervention decays over time and the optimal renewal rate. There could also be head-to-head comparisons with exercise interventions for which the evidence is also favorable and probably stronger [2]. Furthermore, exercise interventions have the additional advantages of providing social contact (for group exercise) and also in terms of chronic disease prevention [26]. Exercise interventions are also able to prevent falls both inside and outside the home environment, whereas HSAM is restricted to the home environment (albeit also potentially benefiting other household members).

Nevertheless, given the results of this study and other evidence collectively (see *Introduction*), HSAM may still be a worthwhile intervention for district level policy-makers to consider. In settings where a health district authority could not readily mobilize resources for a universal HSAM intervention for all those aged 65+, it could still consider options such as:

- Targeting the HSAM intervention to adults aged 65+ or aged 75+ with a prior injurious fall would reduce upfront intervention costs. Such approaches would also provide the opportunity to collect better data on the exact costs and field application of the intervention (e.g., uptake by age-group), before any further scaling up.
- Targeting the HSAM intervention to adults aged 65+ living in rental accommodation or who live in deprived areas. These approaches may potentially contribute health equity benefits.
- Instead of investing in HSAM interventions, health district authorities could work collectively to encourage higher levels of government to instigate a comprehensive national-level HSAM program so as to maximize economies of scale. Alternatively, they could encourage central governments to legislate so that building codes require that all new homes and all rental properties meet minimal safety standards, or that a warrant of fitness program [27] was applied to all existing houses.

In conclusion, this study provides modeling-level evidence that the HSAM intervention may produce considerable health gain and be cost-effective at a health district level. Targeting HSAM to older people with previous injurious falls may even more cost-effective and might be the best place for a resource-constrained health district to start using this intervention. Nevertheless, comparisons may also be desirable with other falls prevention interventions that have additional advantages such as group exercise programs which also provide social contact and may prevent chronic diseases (e.g., as per the association between traditional Chinese exercise in older people and reduced mortality rates [26]).

## Supporting information

S1 Appendix Additional methods and results. (DOCX)

S1 File CHEERS checklist (consolidated health economic evaluation reporting standards). (PDF)
