# Supplementary material for: Home modification to reduce falls at a health district level: Modeling health gain, health inequalities and health costs
Source: PLoS One. 2017 Sep 14;12(9):e0184538. doi: 10.1371/journal.pone.0184538 (PMC5598974; doi:10.1371/journal.pone.0184538)

S1 Appendix: Additional Methods and Results

Figure A. Model structure for studying the home safety assessment and modification (HSAM) intervention in community-dwelling older people at a health district level (Markov macro-simulation model in TreeAge, Figure adapted from Pega et al 2016 – see main manuscript)


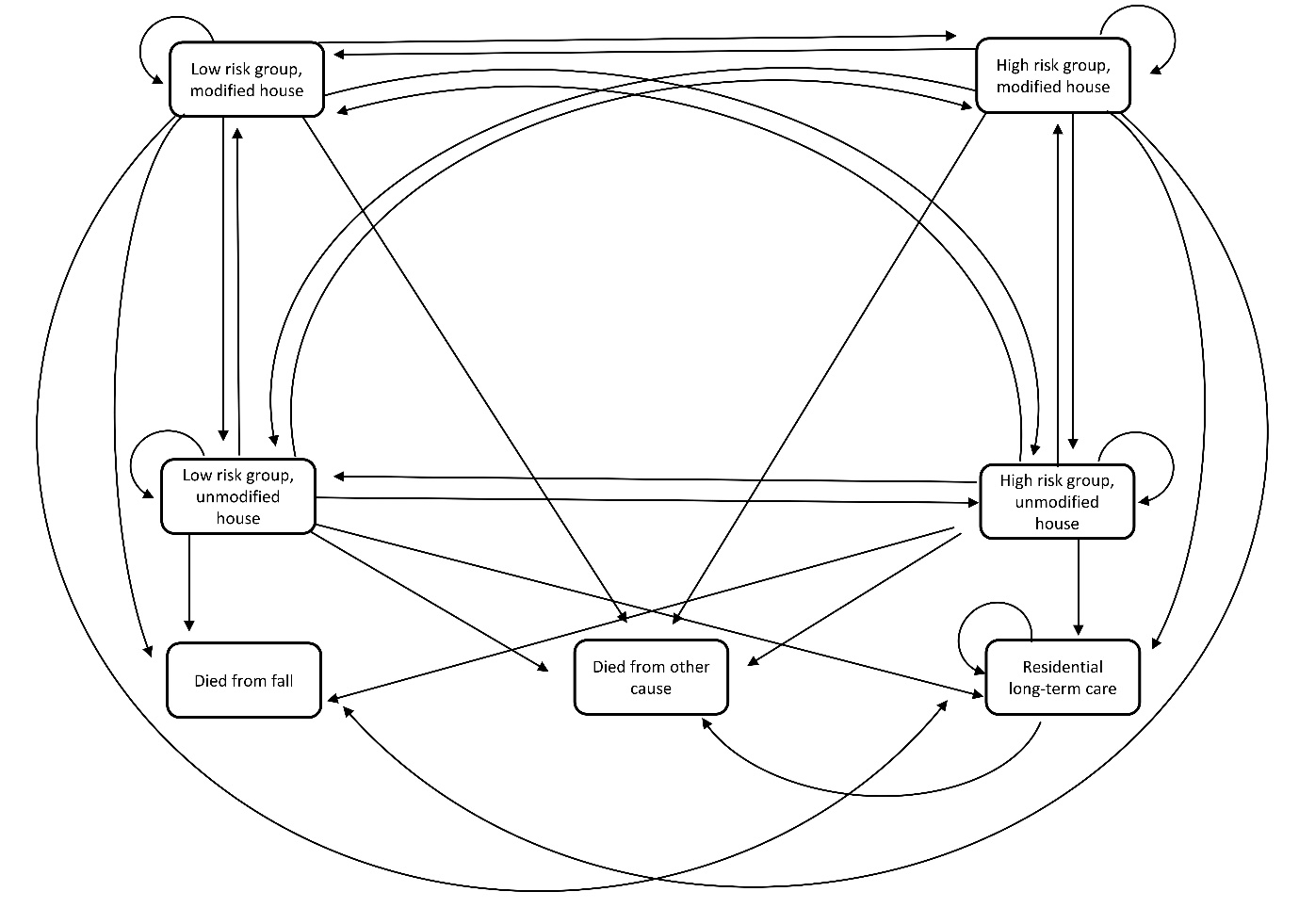


Figure B. Tornado plot for QALYs gained from the HSAM intervention (base-case intervention)


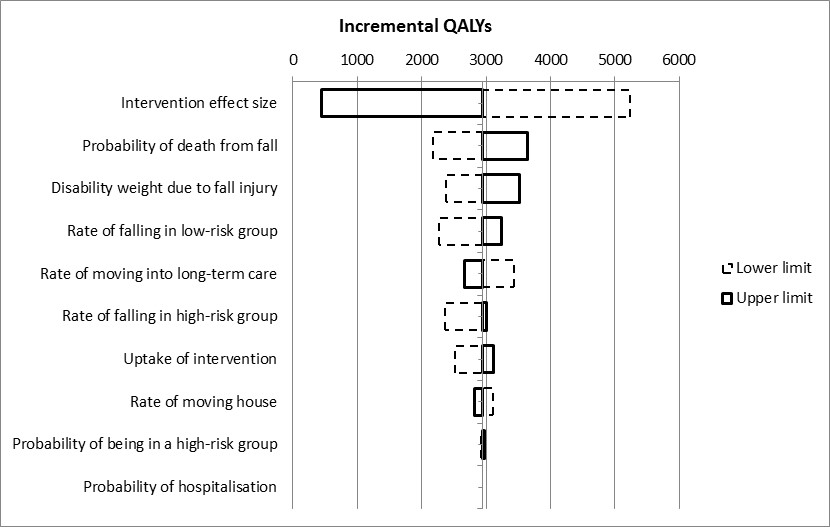


Figure C. Tornado plot for incremental health system costs from the HSAM intervention (base-case intervention)


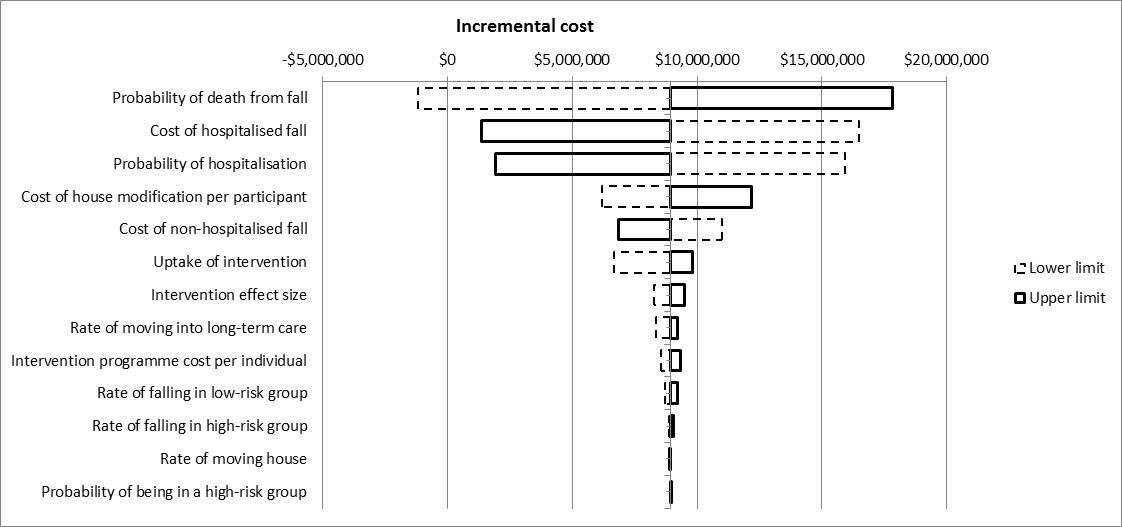


Figure D. Tornado plot for incremental cost-effectiveness ratio (ICER) from the HSAM intervention (base-case intervention)


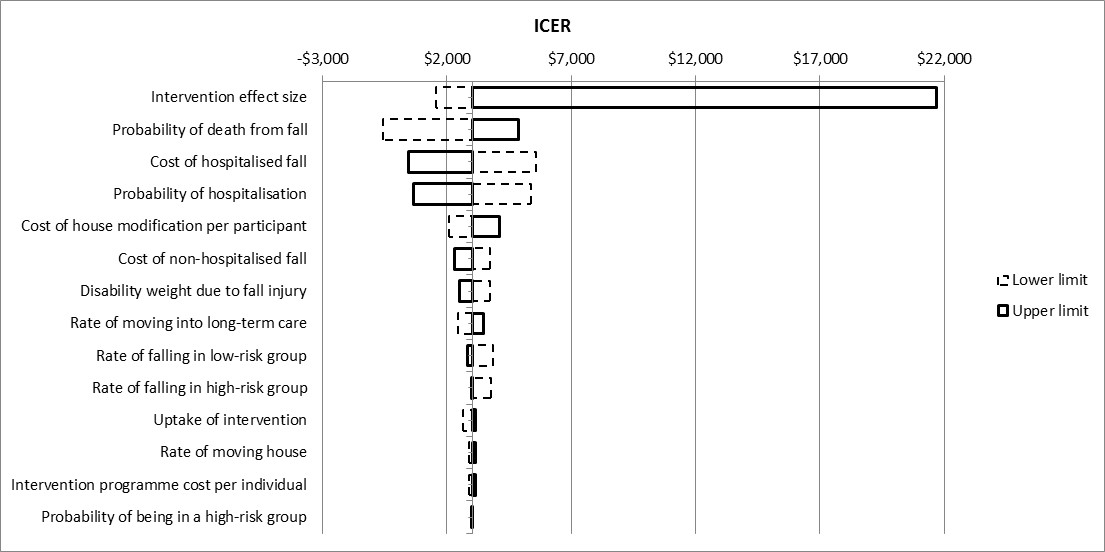

Supplement: S1 Appendix — (DOCX) [file pone.0184538.s001.docx]
